# Molecular Analysis of Two CC-Type Glutaredoxins from the Model Legume *Lotus japonicus*

**DOI:** 10.3390/ijms27156703

**Published:** 2026-07-27

**Authors:** Inmaculada García-Díaz, Antonio Díaz-Quintana, Miguel Roldán, Patricia Gómez-Villegas, Luis López-Maury, Margarita García-Calderón, Antonio J. Márquez, Marco Betti

**Affiliations:** 1 Departamento de Bioquímica Vegetal y Biología Molecular, Facultad de Química, Universidad de Sevilla, C/Prof. García González 1, 41012 Sevilla, Spain; mgdiaz@us.es (I.G.-D.); qzaida@us.es (A.D.-Q.); mroldan@us.es (M.R.); pgomez9@us.es (P.G.-V.); llopez1@us.es (L.L.-M.); marbioq@us.es (M.G.-C.); cabeza@us.es (A.J.M.); 2 Instituto de Investigaciones Químicas, cicCartuja, Universidad de Sevilla-CSIC, C/Américo Vespucio 49, 41092 Sevilla, Spain; 3 Instituto de Bioquímica Vegetal y Fotosíntesis (IBVF), cicCartuja, Universidad de Sevilla-CSIC, C/Américo Vespucio 49, 41092 Sevilla, Spain

**Keywords:** CC-type glutaredoxins, structural modelling, oxidoreductase, recombinant proteins, *Lotus japonicus*

## Abstract

Glutaredoxins (GRXs) are small oxidoreductases involved in redox regulation, but the biochemical properties of plant-specific CC-type GRXs remain poorly understood, particularly in legumes. In a previous transcriptomic analysis, two CC-type glutaredoxins from *Lotus japonicus*, *Lj*GRX460 and *Lj*GRX569, were identified as differentially expressed during symbiosis with nitrogen-fixing rhizobia. Here, both proteins were produced recombinantly and characterized by sequence analysis, structural modelling and in vitro biochemical assays. Phylogenetic and sequence studies confirmed that both proteins belong to the plant-specific CC-type GRX class but differ in active site composition and overall sequence organization. Homology modelling and molecular dynamics simulations revealed distinct conformational properties, including differences in active site accessibility, electrostatic surface distribution and putative oxidation-dependent structural rearrangements. Comparative analyses with representative class I and II GRXs supported substantial structural divergence, suggesting functional specialization. Optimized expression and purification protocols yielded soluble recombinant proteins. Both *Lj*GRX460 and *Lj*GRX569 displayed a strong tendency to form soluble high molecular weight aggregates, similar to the class I control GRX. Enzymatic assays showed low oxidoreductase activity towards classical disulfide substrates compared with class I GRXs, while molecular docking suggested reduced affinity for bis(2-hydroxyethyl) disulfide (HEDS) and preferential interaction with *L*-cystine. These results indicate that *Lj*GRX460 and *Lj*GRX569 are unlikely to function as classical oxidoreductases and may instead perform specialized regulatory functions.

## 1. Introduction

Glutaredoxins (GRXs) were first identified in extracts of *Escherichia coli* lacking thioredoxins (TRXs), in which the presence of a thermostable protein was observed. Due to their ability to use reduced glutathione (GSH) as an electron donor, these proteins were given the name glutaredoxin [1]. They are small proteins, generally comprising between 100 and 200 amino acid residues, belonging to a superfamily that also includes TRXs, glutathione S-transferases, glutathione peroxidases, disulfide bond formation proteins, peroxiredoxins and protein disulfide isomerases. All members of this superfamily are characterized by a TRX-type fold, which consists of a central mixed β sheet of four strands surrounded by three α helices [2], although the number of α helices and the number of strands in the β sheet may vary slightly among them [3]. Another common feature of the proteins within this superfamily is the presence of an active site composed of a four amino acid motif. The first of these residues is a conserved cysteine that protrudes from the protein surface, which is essential for redox reactivity [3].

**Scheme 1:**
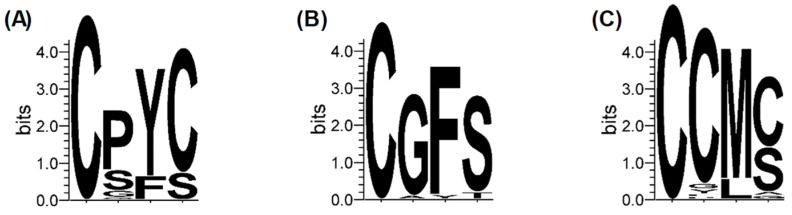
**GRX classification**. Sequence logo of the active site in each GRX class ((**A**) class I; (**B**) class II; (**C**) class III) performed with WebLogo v3 [7] using data available from *Escherichia coli*, *Trypanosoma brucei*, *Synechocystis* sp. PCC 6803, *Chlamydomonas reinhardtii*, *Saccharomyces cerevisiae*, *Homo sapiens*, *Arabidopsis thaliana*, *Oryza sativa* and *Populus trichocarpa*.

Depending on the characteristics of the active site, which is located after the first β strand, different types of GRXs can be established (Scheme 1). Although some CXXX-type members can be found in class I GRXs, they generally have a CXXC-type active site. These include sequences such as CGYC, CSY[C/S], CPFC and CPYC, with the latter being the most frequent and, therefore, leading to the alternative designation of this group as the CPYC group (Scheme 1A). From a biochemical perspective, these GRXs possess conserved GSH binding regions [4] and are well characterized. They have been mainly associated with redox homeostasis and regulation through their activity as oxidoreductases or thioltransferases in a variety of biological processes [5,6].

Class II GRXs, also known as the CGFS group, possess a highly conserved active site, usually represented by the CGFS motif, with a single cysteine (Scheme 1B). Most of these proteins are mainly associated with the assembly and transfer of Fe–S clusters [8]. In fact, they participate in the biogenesis of Fe–S centers, most frequently as [2Fe–2S] cluster-binding proteins, although binding to [3Fe–4S] or [4Fe–4S] clusters has also been proposed [9]. Moreover, they can transfer these Fe–S centers to target apoproteins, either directly or through heterocomplexes [8], thereby playing a central role in cellular iron metabolism and homeostasis.

Class I and class II GRXs can be found in both prokaryotic and eukaryotic organisms; however, class III (known as ROXYs) comprises a particular group of GRXs that occur exclusively in plants [6]. The main characteristic of these GRXs, also named CC-type GRXs, is that, with a few exceptions [10], they contain two contiguous cysteine residues, occupying the first two positions of the active site (CCXX) (Scheme 1C). This active site can be the CCXX or CCXC type if a third cysteine occupies the fourth position.

Despite recent advances, several aspects of ROXY functions in higher plants remain unknown. It is not clear whether the redox enzymatic activity of plant CC-type GRXs is essential for their function in vivo [11,12]. Importantly, further progress on the basic biochemistry of class III GRXs has been hampered by the difficulties in producing soluble recombinant proteins [5]. In legumes, the role of these GRXs remains even less understood, although they have been proposed to participate in redox-dependent post-translational modifications, and their expression has been shown to respond to abiotic stress [13]. Recently, we identified several CC-type GRXs that were differentially expressed in *Lotus japonicus* plants grown in symbiosis with *Mesorhizobium loti* compared to non-symbiotic conditions [14]. In the present work, we further analyze at the molecular level two of these proteins, named *Lj*GRX460 and *Lj*GRX569 according to the last three digits of their gene ID code from the v3.0 *L. japonicus* genome version of the MG20 accession. These proteins have been recombinantly expressed and purified from *E. coli* and subsequently characterized by sequence analysis, three-dimensional modelling, redox activity assays, and substrate interaction studies. The results obtained indicate that these two symbiosis-responsive CC-type GRXs may perform functions distinct from canonical class I glutaredoxins, potentially involving non-canonical redox interactions or protein regulatory mechanisms.

## 2. Results

### 2.1. Comparative Analysis of GRX Protein Sequences

**Scheme 2:**
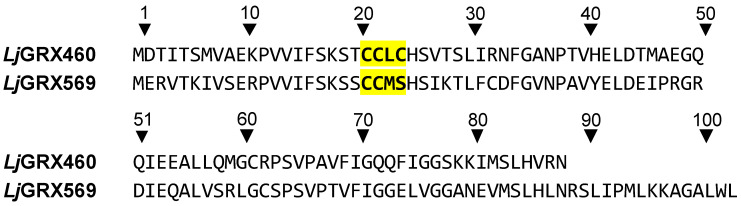
***Lj*GRX460 and *Lj*GRX569 protein sequences**. Deduced amino acid sequences for *Lj*GRX460 and *Lj*GRX569. The four residues of the active site are highlighted in bold letters on a yellow background.

*Lj*GRX460 and *Lj*GRX569 possess an active site with two contiguous cysteine residues, a defining feature of class III GRXs (Scheme 2). *Lj*GRX460 contains a third cysteine at the fourth position of the active site motif (CCXC), resembling the class I motif, whereas *Lj*GRX569 has a serine at this position, making it more similar to class II GRXs (CCXX). The two proteins also exhibit distinct sequence lengths, with *Lj*GRX569 being longer than *Lj*GRX460 (Scheme 2).

**Figure 1:**
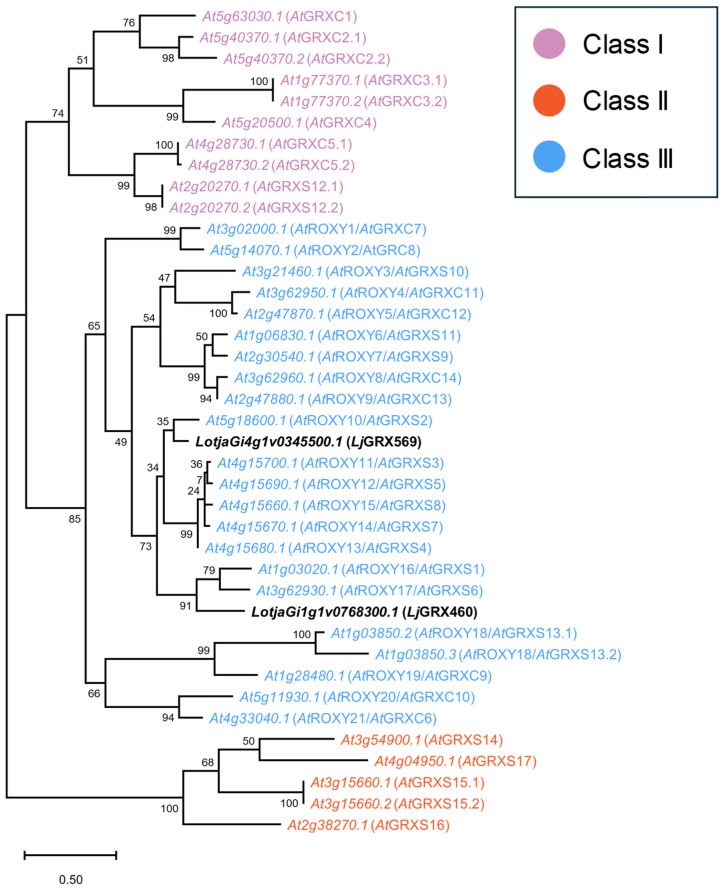
***Lj*GRX460 and *Lj*GRX569 phylogenetic analysis**. Phylogenetic tree inferred from the complete amino acid sequences of the GRXs of *A. thaliana*, *Lj*GRX460 and *Lj*GRX569 (the last two indicated in bold letters), using the Maximum Likelihood method and Le–Gascuel (2008) model [15] of amino acid substitutions. The percentage of replicate trees in which the associated taxa clustered together (1000 replicates) is shown next to the branches. For each GRX, the ID transcript and common name(s) are shown in parenthesis. Violet, orange and blue indicate *At*GRXs belonging to class I, II and III, respectively.

Phylogenetic analysis of *Lj*GRX569 and *Lj*GRX460 alongside the known *Arabidopsis thaliana* GRXs revealed that their closest *A. thaliana* homologues based on both phylogenetic reconstruction and sequence identity analyses are *At*ROXY10 and *At*ROXY16/*At*ROXY17, respectively (Figure 1; Table S1), which correspond to relatively uncharacterized *At*ROXY proteins. Notably, the phylogenetic relationships shown in Figure 1 suggest that class I and class III GRXs may share a common ancestor distinct from class II GRXs.

**Figure 2:**
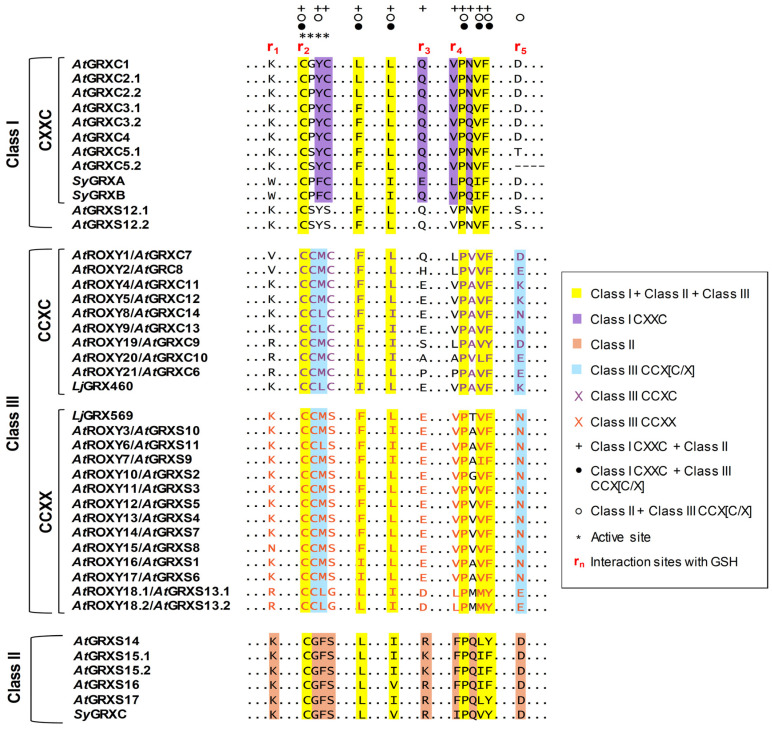
**Alignment and conservation analysis of protein sequences from GRXs of different classes and species**. Multiple sequence alignment of the most conserved regions of the GRXs from *A. thaliana* and from *Synechocystis* sp. PCC 6803, *Lj*GRX460 and *Lj*GRX569, performed with Clustal Omega v1.2.4 [16]. Yellow, violet, orange or blue background indicate residues that are conserved in all GRXs analysed, in class I CXXC type and in class II or class III CCX[C/X] type. Similarly, violet letters indicate residues that are conserved in class III CCXC-type GRXs, and orange letters indicate residues that are conserved in class III CCXX-type GRXs. Crosses, black circles and unfilled circles indicate residues that are conserved between class I CXXC type and class II, between class I CXXC type and class III CCX[C/X] type, and between class II and class III CCX[C/X], respectively. Residues of the active site are indicated with asterisks, and the positions of residues that have been proposed to contact with GSH according to Mrozek et al. [17] are marked as r_1–5_.

Figure 2 shows the most conserved regions found for *Lj*GRX460 and *Lj*GRX569 in comparison to all the GRXs described for *A. thaliana* (*At*GRX) and *Synechocystis* sp. PCC 6803 (*Sy*GRX), as representatives of eukaryotic and prokaryotic photosynthetic organisms. The full multiple sequence alignment is shown in Figure S1. This analysis revealed only six residues strictly conserved across all analyzed GRXs. These residues correspond to (i) the first cysteine of the active site; (ii) a leucine, phenylalanine or isoleucine located ten residues downstream; (iii) a leucine positioned twelve residues further downstream, occasionally substituted by a isoleucine or a valine; (iv) a highly conserved proline near the C-terminal region; and the residues located two and three positions downstream of this proline, usually represented by (v) a valine and (vi) a phenylalanine, respectively. The conservation of these residues (highlighted with yellow background in Figure 2) suggests that they are likely to play an important structural role.

Despite differences between the CCXX and CCXC subtypes, most class III GRXs harbor a C-terminal GALWL-like motif, seen in *Lj*GRX460, *Lj*GRX569 and other *At*GRXs, regardless of their specific subtype (Figure S1). Furthermore, only two out of the five residues proposed by Mrozek et al. [17] to participate in GSH binding (indicated as r_1–5_ in Figure 2) were conserved in CC-type GRXs. These correspond to the cysteine located at the first position of the active site (r_2_) and the residue designated as r_5_. In most class I and II GRXs, r_5_ is an aspartate, whereas in class III GRXs this position is commonly occupied by an asparagine, as observed in *Lj*GRX569, although some proteins contain a glutamate or, as in *Lj*GRX460, a lysine. The remaining three residues showed marked variability among GRX classes. Residue r_1_ generally corresponds to a lysine located upstream of the active site, at a position that varies depending on the GRX group (Figure S1). Residue r_3_ is typically an arginine or lysine in class II GRXs, whereas class I proteins usually contain a conserved glutamine at this position. An exception was *Sy*GRXA, which presented a glutamate, the residue most frequently found in class III GRXs, particularly in the CCXX subtype. Finally, residue r_4_, located immediately before the conserved C-terminal proline, is generally a valine in class I GRXs and a phenylalanine in class II proteins, whereas in ROXYs it can appear as a valine, a leucine, an alanine or a proline. These differences indicate a substantial structural divergence among GRX classes that likely contributes to their distinct biochemical properties and biological functions.

### 2.2. Comparative Structural Modelling Reveals Divergent Active Site Architecture

**Figure 3:**
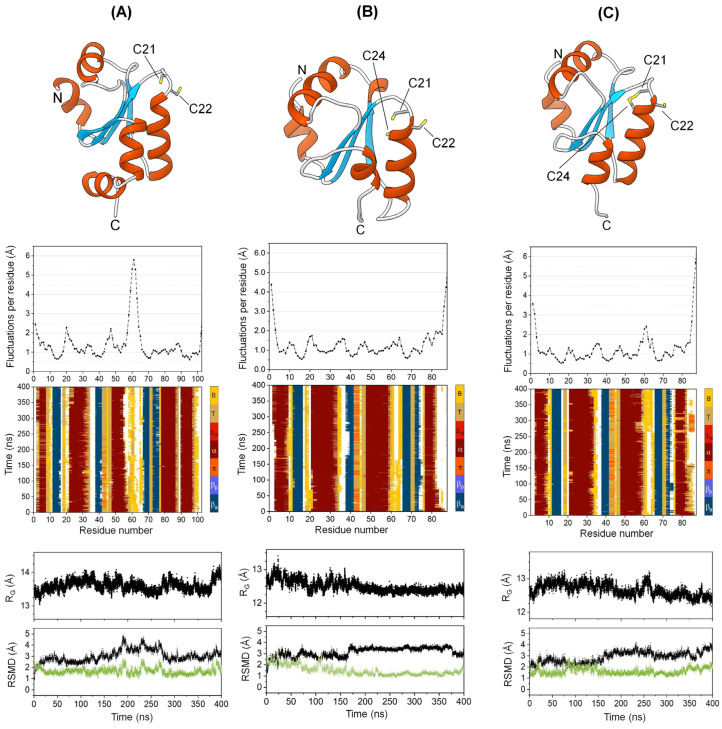
**Structural models of *Lj*GRX monomers and global statistics calculated through 400 ns of molecular dynamics**. For each model ((**A**) reduced *Lj*GRX569 monomer; (**B**) reduced *Lj*GRX460 monomer; (**C**) oxidized *Lj*GRX460 monomer with an intramolecular disulfide between C21 and C24), a Richardson diagram is shown, obtained from the coordinates of the structure closest to the average of the last 350 ns of the trajectory, optimised by energy minimisation, the atomic fluctuations per residue and the evolution of the protein secondary structure elements along the trajectory and calculated using the dictionary of protein secondary structure (DSSP) algorithm [18] as well as the protein radius of gyration (R_G_) and root mean square deviation (RMSD) of the backbone coordinates relative to the initial structure as optimised by energy minimisation (black) and to the average structure of the last 350 ns (green). Abbreviations of secondary structure elements: β (Isolated β-bridge), T (hydrogen-bonded turn), 3_10_ (3_10_-helix), α (α-helix), π (π-helix), β_ρ_ (left-handed k-helix), β_a_ (extended strand, participates in β_-_ladders).

To correlate these sequence features with protein structure, and because no class III GRX has yet been structurally characterized by X-ray crystallography, electron microscopy or nuclear magnetic resonance, three-dimensional models of the *Lj*GRX monomers were generated by homology modelling. These models were subsequently refined through energy minimization followed by molecular dynamics simulations, averaging coordinates from a selected stable part of the trajectory, followed by a final energy minimization. Using this approach, a structural model of the *Lj*GRX569 monomer was obtained, consisting of five α-helices arranged around four central β-strands (Figure 3A). The *Lj*GRX569 monomer displayed a relatively compact structure, with a 13.6 ± 0.16 Å radius of gyration, but also exhibited fluctuations of the plateau values (3.2 ± 0.49 Å) of the root mean square deviation (RMSD) of the backbone coordinates with respect to the starting structures, reflecting substantial conformational flexibility (Figure 3A). The model showed high fluctuations around residue 60 (Figure 3A), indicating a high flexibility of the loop connecting the third α-helix and the third β-strand.

Initially, it was hypothesized that this flexibility could result from interactions between the two cysteine residues located at the beginning of the active site, potentially leading to the formation of an intramolecular disulfide bond near the highly fluctuating region. However, although these cysteines, located immediately upstream of the second α-helix, occupy contiguous positions, their side chains project in opposite directions relative to the peptide backbone (Figure 3A). Consequently, the inter-sulfur distance between the corresponding thiol groups was relatively large (6.267 Å; Figure S2A), rendering intramolecular disulfide bond formation unfeasible. Attempts to reduce this distance through rotational adjustments of the side chains were unsuccessful. Therefore, only the reduced state structure of the monomer was considered for this protein. In contrast, both the reduced state (Figure 3B) and an oxidized state containing a C21–C24 intramolecular disulfide bond (Figure 3C) were modelled for the *Lj*GRX460 monomer. Formation of this bond appeared structurally feasible because the additional cysteine located at the end of the active site motif, positioned its sulfur atom within 3.627 Å of the C21 sulfur atom (Figure S2B). Furthermore, the angle between the β-carbon atoms was approximately 90° (Figure S2B), a geometry highly compatible with disulfide bond formation. However, analogous to the *Lj*GRX569 monomer, the sulfur atoms of C21 and C22, occupying the first two positions of the active site, remained separated by more than 6 Å (Figure S2B).

Comparison of the oxidized and reduced *Lj*GRX460 structural models revealed that, unlike *Lj*GRX569, both forms contained only three central β-strands (Figure 3B,C). In addition, the number of α-helices surrounding these β-strands differed depending on the oxidation state. Whereas the reduced form contained five α-helices, only four were observed in the oxidized form (Figure 3B,C). This missing α-helix corresponds to a short segment located downstream of the second β-strand in the reduced structure. Notably, this region did not coincide with the area showing the greatest structural fluctuation, which instead mapped to the C-terminal α-helix (Figure 3B,C). The reduced state model of *Lj*GRX460 exhibited greater residue fluctuation than the oxidized state model, whereas RMSD values were broadly similar but slightly lower in the oxidized form (reduced state: 3.27 ± 0.64 Å; oxidized state: 2.95 ± 0.47 Å; Figure 3B,C). These observations suggest that the oxidized form may represent a more structurally stable state of *Lj*GRX460. In this state, the protein adopted a structure with an average radius of gyration equal to 12.67 ± 0.19 Å (Figure 3C), as expected from its smaller sequence and being somewhat more compact than that of *Lj*GRX569 (Figure 3A).

**Figure 4:**
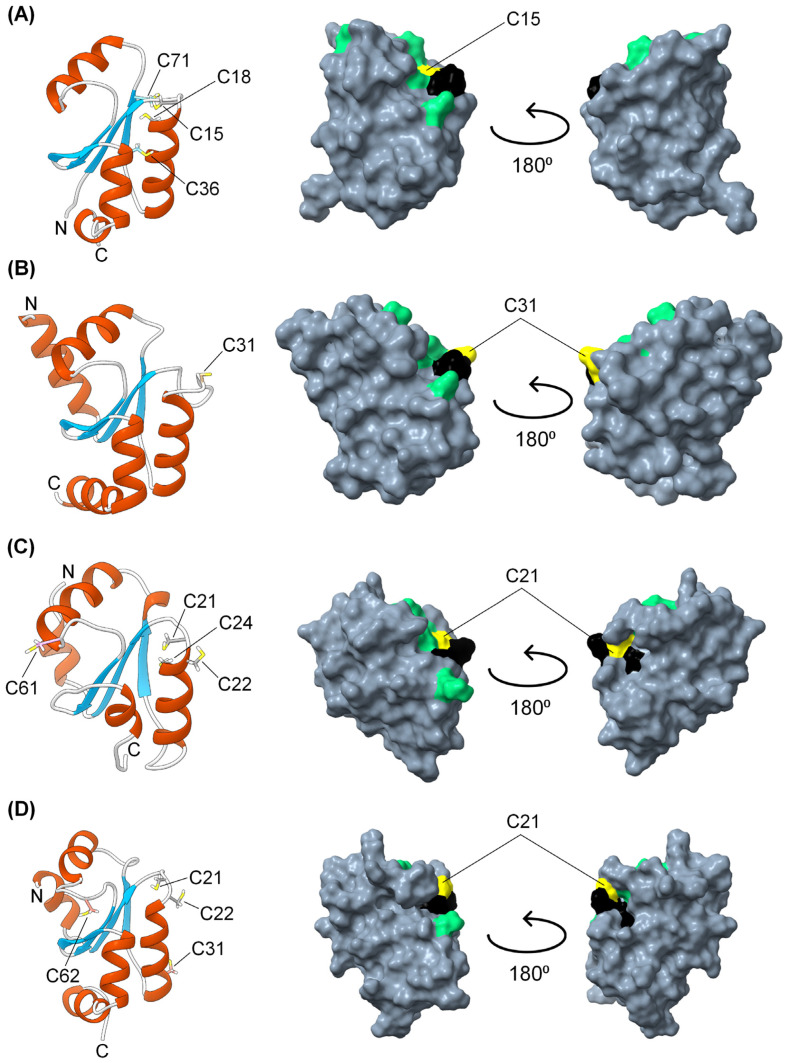
**Models of reduced GRX monomers**. For each GRX ((**A**) *Sy*GRXA; (**B**) *Sy*GRXC; (**C**) *Lj*GRX460; (**D**) *Lj*GRX569) the model of the secondary (left) and tertiary (right) structures obtained with Chimera v1.19 software is shown [20]. In the secondary structures, all the cysteine residues are indicated. In the surface models, the first cysteine of the active site is shown in yellow, the other three residues of the motif in black, and residues r_1–5_ in Figure 2 in green.

To obtain a broader structural comparison across GRX classes, the structures of *Sy*GRXA and *Sy*GRXC monomers from *Synechocystis* sp. PCC 6803, representing class I and class II GRXs, respectively, were also analyzed. For *Sy*GRXA, the structure derived from protein data bank (PDB) entry 3QMX, corresponding to the X-ray crystal structure reported by Kim et al. [19], was used. In contrast, the *Sy*GRXC structure was generated by homology modelling. *Sy*GRXA exhibited several distinctive features compared with *Sy*GRXC and the two *L. japonicus* class III GRXs (Figure 4). First, *Sy*GRXA lacked the N-terminal α-helix, and the side chains of its active site cysteines were oriented towards the interior of the structure (Figure 4A). This contrasted with *Sy*GRXC, in which the thiol group of the single active site cysteine was oriented towards the solvent-exposed surface (Figure 4B), similarly to the cysteine occupying position 22 in *Lj*GRX460 and *Lj*GRX569 (Figure 4C,D). Surface representations derived from these structural models further highlighted differences among GRX classes, particularly within the active site region. As shown in Figure 4A, the active site of *Sy*GRXA adopted a groove-like conformation in which the first active site cysteine was located in the center. In *Sy*GRXC, the equivalent cysteine was more solvent-exposed, and the active site appeared sterically restricted (Figure 4B). Regarding *Lj*GRX460 and *Lj*GRX569, the active site region was considerably less accessible (Figure 4C,D), suggesting that these proteins may show substrate interaction properties distinct from those of canonical oxidoreductase GRXs.

**Figure 5:**
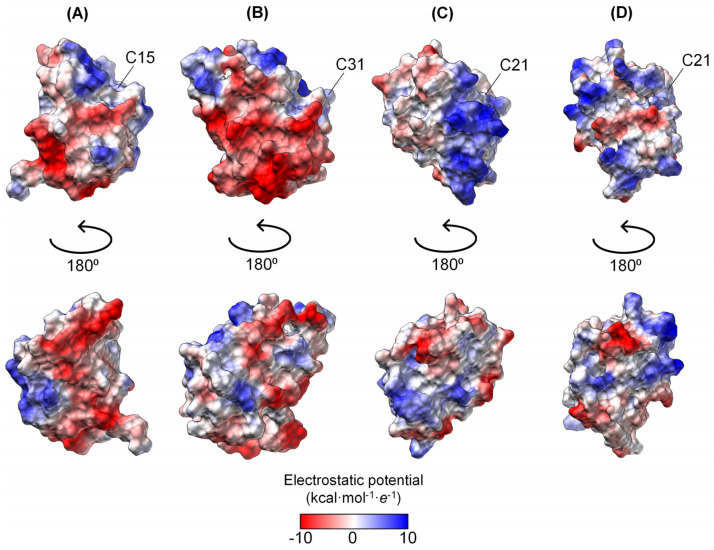
**Electrostatic potential of reduced GRX monomers**. For each GRX ((**A**) *Sy*GRXA; (**B**) *Sy*GRXC; (**C**) *Lj*GRX460; (**D**) *Lj*GRX569) the electrostatic potential on the surface, determined using Chimera v1.19 software, is shown [20]. The first cysteine of the active site is indicated.

Differences among GRX groups were not limited to structural organization, as variations in surface electrostatic properties were also observed. Electrostatic surface potential analysis revealed that the *Sy*GRX monomers predominantly displayed regions of negative electrostatic potential (Figure 5A,B), whereas the *Lj*GRX monomers showed more positively charged surfaces (Figure 5C,D), particularly on the face containing the active site. In *Lj*GRX569, positively charged regions surrounded a central negatively charged zone (Figure 5D), potentially generating a quadrupolar electrostatic distribution. In contrast, *Lj*GRX460 displayed more clearly separated regions of opposite electrostatic potential, forming a dipole-like distribution (Figure 5C).

*Sy*GRXA and *Sy*GRXC also showed dipole regions (Figure 5A,B), although in the latter, the distribution appeared to be more shielded. Hydrophobicity analyses revealed broadly similar profiles among all GRXs examined. However, *Lj*GRX monomers (Figure S3A,B) seemed slightly more hydrophobic than *Sy*GRXC (Figure S3C) and *Sy*GRXA (Figure S3D) monomers, with the latter representing the most hydrophilic protein analyzed. Although these differences were less pronounced than those observed for structural or electrostatic properties, they may nevertheless contribute to the distinct functional behavior of each GRX class.

### 2.3. Production of Recombinant Proteins and Analysis of Molecular Properties

**Figure 6:**
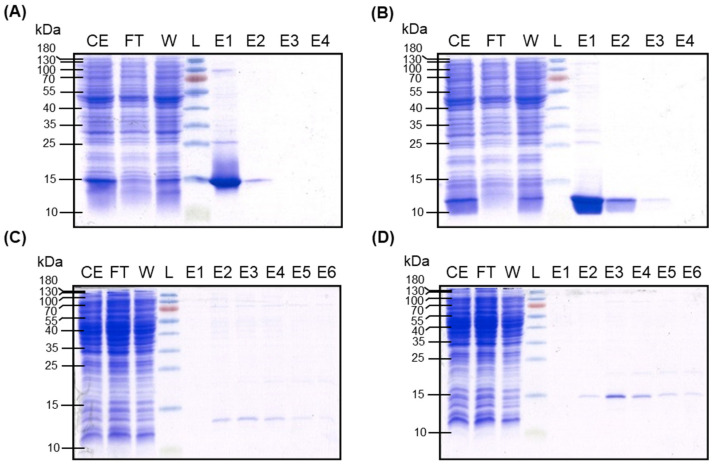
**Purification of recombinant GRXs**. Representative 15% (*w*/*v*) acrylamide SDS-PAGE gels showing samples from each of the fractions (CE: crude extract; FT: Flow through; W: washes, L: ladder; E: eluates) obtained during the purification process of the different recombinant GRXs ((**A**) *Sy*GRXA; (**B**) *Sy*GRXC; (**C**) *Lj*GRX460; (**D**) *Lj*GRX569).

To investigate the molecular characteristics of the *Lj*GRXs of interest, the corresponding proteins were produced in recombinant form together with the two *Sy*GRXs used above as representatives of class I and II GRXs (Figure 6). *Sy*GRXA and *Sy*GRXC were purified as N-terminal 6xHis fusion proteins (Figure 6A,B). Attempts to purify *Lj*GRX460 and *Lj*GRX569 as His-tagged proteins were unsuccessful because of the very low solubility of the recombinant proteins under all the conditions tested. Consequently, an alternative strategy using a Strep-tag II fusion was deployed. Larger solubility tags were avoided to prevent potential structural artifacts. Following extensive optimization of induction times and temperatures, isopropyl β-D-1-thiogalactopyranoside (IPTG) concentration, optical density at 600 nm (OD_600_) and culture volume, the purification of soluble *Lj*GRX460 and *Lj*GRX569 was achieved (Figure 6C,D).

After purification, ultrafiltration was performed to concentrate *Lj*GRX460 and *Lj*GRX569 and improve sample purity. However, it was found that none of these proteins passed through 20 kDa molecular weight cut-off membranes, suggesting that both formed high molecular weight species in solution. Subsequent use of filters with different molecular weight cut-offs revealed that virtually the entire protein sample remained in the fraction corresponding to molecular weights greater than 100 kDa.

**Figure 7:**
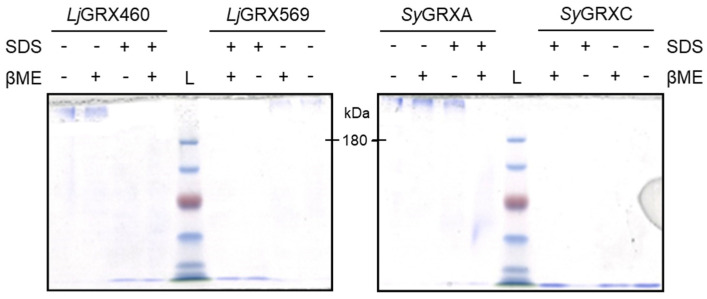
**Analysis of recombinant GRX oligomerisation by non-denaturing electrophoresis**. Samples of recombinant *Lj*GRX460, *Lj*GRX569, *Sy*GRXA and *Sy*GRXC were incubated with loading buffers containing different combinations of 1% (*w*/*v*) SDS and 2.5% (*v*/*v*) β-mercaptoethanol (βME) and subsequently subjected to non-denaturing electrophoresis on native 8% (*w*/*v*) acrylamide gels. L: PageRuler^TM^ Prestained Protein Ladder (ThermoFisher Scientific, Alcobendas, Madrid, Spain) prepared in loading buffer containing 1% (*w*/*v*) SDS and 2.5% (*v*/*v*) β-mercaptoethanol, with molecular weights, from highest to lowest, of 180, 130, 100, 70, 55, 40, 35, 25, 15 and 10 kDa.

The native polyacrylamide gel electrophoresis (PAGE) of purified protein samples was carried out with or without previous incubation with sodium dodecyl sulfate (SDS) and/or β-mercaptoethanol (Figure 7).

In the absence of SDS, both *Lj*GRX460 and *Lj*GRX569 formed soluble high molecular weight aggregates that were unaffected by β-mercaptoethanol. However, in samples treated with SDS, these aggregates dissociated, suggesting that subunit interactions are non-covalent. Notably, a similar tendency to form aggregates was observed for *Sy*GRXA but not for *Sy*GRXC. Complete dissociation of *Sy*GRXA aggregates, however, required the synergistic action of both SDS and β-mercaptoethanol (Figure 7), suggesting that disulfide bonds may contribute to the aggregation of this specific class I isoform.

### 2.4. L. japonicus CC-Type GRXs Show Limited Oxidoreductase Activity

**Figure 8:**
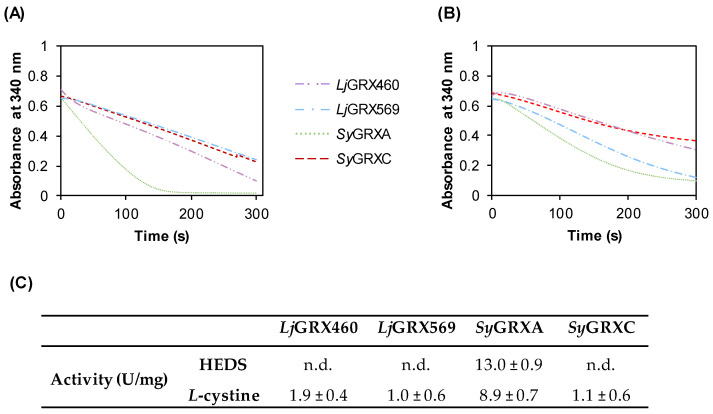
**Oxidoreductase activity assays of recombinant GRXs with different disulfides**. Absorbance curves at 340 nm using HEDS (**A**) or *L*-cystine (**B**) as substrate. (**C**). Enzymatic activity obtained in the assays using two technical replicates (n.d.: non-detected).

Under the conditions tested, *Sy*GRXA was the only one of the four GRX proteins analyzed to produce clear oxidoreductase activity towards bis(2-hydroxyethyl) disulfide (HEDS) (Figure 8A–C). However, when HEDS was replaced by *L*-cystine, activity was detected not only for *Sy*GRXA but also for *Lj*GRX460, *Lj*GRX569 and *Sy*GRXC (Figure 8B,C). However, the activity of these last three proteins towards *L*-cystine was markedly lower than that of *Sy*GRXA. Overall, the oxidoreductase assays indicate that *Lj*GRX460 and *Lj*GRX569 do not show typical disulfide oxidoreductase activity.

### 2.5. Molecular Docking Analysis of GRX–Substrate Interactions

To further investigate the potential catalytic properties obtained for the analyzed GRXs, molecular docking studies were performed using the monomeric protein models generated. *Sy*GRXA, which displayed the highest oxidoreductase activity (Figure 8), and *Lj*GRX460, which among the less active GRXs presented the most similar active site organization to that of *Sy*GRXA, were selected as reference proteins for these analyses. Initially, blind docking was carried out to evaluate the interaction of each structural model with HEDS. The results revealed a greater number of docking solutions located near the first active site cysteine in *Sy*GRXA than in *Lj*GRX460, suggesting a higher probability of substrate interaction within the catalytic region of *Sy*GRXA. This observation may provide a structural rationale for the higher catalytic activity experimentally observed for this protein.

Subsequently, directed docking towards the active site was performed using both HEDS and *L*-cystine. The resulting docking poses were grouped into clusters according to geometric similarity, using an RMSD cut-off value of 2 Å for heavy atoms. The corresponding mean dissociation constants and binding energies are summarized in Table 1. For each protein–substrate combination, the cluster displaying the lowest estimated binding energies was selected for further analysis (Table 1).

Comparison of the parameters associated with these conformations showed that the interaction between *Lj*GRX460 and HEDS was predicted to be less stable than that observed for *Sy*GRXA, as indicated by the less favorable binding energy values. A similar trend was observed for *L*-cystine (Table 1). In both proteins, the predicted dissociation constants obtained for *L*-cystine were markedly lower than those calculated for HEDS, particularly in *Sy*GRXA, where the difference exceeded three orders of magnitude. These results suggest that both proteins exhibit higher affinity for *L*-cystine than for HEDS. The observed differences appear to be associated mainly with electrostatic interactions, as the electrostatic energy term was the one showing the greatest variation between substrates (Table 1).

## 3. Discussion

CC-type GRXs, also known as ROXYs, remain the least biochemically characterized class of GRXs. In the present study, two CC-type GRXs from *L. japonicus*, *Lj*GRX460 and *Lj*GRX569, previously identified as symbiosis-responsive proteins, were successfully expressed and purified in soluble form. This enabled their comparative biochemical characterization. Previous studies have highlighted the difficulty of obtaining soluble recombinant plant CC-type GRXs, which has limited direct analyses of their catalytic and structural properties. Couturier et al. [21], for example, attempted to express several class III GRXs from *Populus trichocarpa* in different *E. coli* strains but obtained low expression levels for all proteins analyzed. More recently, strategies based on proteins fused to tags substantially larger than the protein itself have been employed to improve the solubility of *A. thaliana* and *P. trichocarpa* GRXs [10,17]. A Strep-maltose-binding protein (MBP) fusion construct using a baculovirus system enabled the production of soluble *At*ROXY9 [17]. Similarly, better yields were reported for the purification of 22 CC-type GRX isoforms from *P. trichocarpa* as N-terminal MBP-tag fusion proteins lacking the first 12 amino acids corresponding to a predicted signal peptide. In contrast, equivalent constructs containing glutathione S-transferase (GST) or 6×His tags produced insoluble proteins or proteins that remained unstable and precipitated during purification [10]. In the present work, soluble recombinant *Lj*GRX460 and *Lj*GRX569 were successfully produced as proteins fused to a small N-terminal Strep-tag II, although this required low temperature induction and extensive optimization of expression and purification conditions. These results provide a useful methodological framework that may facilitate future biochemical studies of class III GRXs.

A major objective of the present study was to determine whether the *L. japonicus* class III CC-type GRXs have redox catalytic activity. To address this question, *Lj*GRX460 and *Lj*GRX569 were analyzed comparatively with two *Synechocystis* sp. PCC 6803 GRXs representing class I and class II proteins, *Sy*GRXA and *Sy*GRXC, respectively. The enzymatic assays revealed substantial functional divergence among the analyzed proteins. As expected, the class I GRX displayed oxidoreductase activity towards HEDS, whereas no detectable activity with this substrate was observed for *Sy*GRXC, *Lj*GRX460 or *Lj*GRX569. However, when *L*-cystine, a molecule that resembles the redox active moiety of oxidized glutathione (GSSG), was used as substrate, measurable activity was detected for both *L. japonicus* proteins and for *Sy*GRXC (Figure 8). These findings represent an advance in the biochemical characterization of GRXs, since recent studies have failed to detect significant oxidoreductase activity towards disulfides for class III GRXs [10,17].

Comparison of the catalytic properties of *Lj*GRX460 and *Lj*GRX569 with previously characterized GRXs further supports the conclusion that these proteins are unlikely to function as classical oxidoreductases. Class I GRXs from diverse organisms, including *E. coli*, *S. cerevisiae*, *C. reinhardtii*, *A. thaliana* and *P. trichocarpa*, generally display readily detectable HEDS-dependent oxidoreductase activity, with reported turnover numbers ranging from a few s^−1^ to several hundred s^−1^ depending on the enzyme and assay conditions [10,22,23,24,25,26]. The turnover number of *Sy*GRXA towards HEDS measured in this work can be estimated from its specific activity to be around 2.0 s^−1^, which falls within the lower end of the range of class I GRX activity. By contrast, the class III GRXs characterized so far consistently exhibit little or no detectable activity in this assay. For example, no HEDS-dependent activity was detected for the *P. trichocarpa* CC-type glutaredoxins *Pt*GRXA1–27 [10] or for *At*ROXY9 [17]. Likewise, neither *Lj*GRX460 nor *Lj*GRX569 catalyzed HEDS reduction under the conditions tested in the present study, while both displayed only very low activity towards *L*-cystine. Although direct quantitative comparisons between studies should be interpreted with caution because of differences in substrates and assay conditions, the available biochemical evidence consistently indicates that class III GRXs diverge from the canonical oxidoreductase function that characterizes class I GRXs. Instead, these proteins are increasingly recognized as specialized regulatory factors whose biological roles are likely mediated through specific protein–protein interactions and redox-dependent modulation of target proteins rather than through efficient thiol-disulfide oxidoreductase activity.

To better understand the structural basis underlying these biochemical differences, we next compared the predicted three-dimensional organization of the four GRXs analyzed. Homology modelling of protein monomers provided additional clues to explain the low oxidoreductase activity of class II and III GRXs. A more sterically hindered and less accessible active site was observed in the *L. japonicus* GRXs (Figure 4). In addition, the structural differences among GRX classes suggest that catalytic activity may be also influenced by specific loop regions surrounding the active site. The loop formed by the residues between the first active site cysteine and the first GSH binding residue (r_1_ and r_2_ of Figure 2) determines the location of that cysteine. In class II GRXs, this loop is longer and more exposed, placing the lysine (r_1_) farther from the catalytic cysteine. However, because ROXY GRXs display a shorter loop arrangement, similar to class I, but still show very low activity, this feature alone cannot explain their lower redox catalytic activities. The position of an Ω-loop located upstream the third strand of the β sheet could be modulated by the N-terminal α-helix, and it might also influence active site architecture. Consistent with this idea, *Sy*GRXA, which showed the highest catalytic activity, was the only protein analyzed lacking this N-terminal α-helix, suggesting that the reduced redox activity of CC-type GRXs may result from the combined effect of several structural features rather than a single determinant. In fact, this Ω-loop surrounding the conserved proline adjacent to r_5_ (in Figure 2) forms a flexible structural element frequently involved in ligand recognition [27,28]. It is noteworthy that in the models generated in the present work, this region showed high flexibility (Figure 3), particularly in *Lj*GRX569. The residue immediately preceding that proline may influence the pK_a_ and reactivity of the catalytic cysteine [29]. The reactivity of the first cysteine residue of the active site could also have an impact on the differences in enzyme activity detected among the different GRXs. Although the pK_a_ of this residue is low in all of them, class II and III GRXs usually have slightly higher pK_a_ values than class I GRXs [17]. Likewise, in CXXC-type GRXs, the pK_a_ of the first cysteine is also lower than that of the cysteine located two positions downstream [30], which could contribute to stabilization of the reaction intermediate according to the cysteine resolution model [31]. This could occur in *Sy*GRXA, which, in addition to the two cysteines of the active site, has two other cysteines that are distant in the primary structure but spatially close to the catalytic region in three-dimensional space (Figure 4).

The docking analyses carried out were consistent with the biochemical analyses carried out. Compared with *Sy*GRXA, *Lj*GRX460 showed a weaker interaction with HEDS within the active site region, with a substantially higher predicted dissociation constant for this substrate (Table 1). Targeted docking also predicted a more favorable interaction between *Lj*GRX460 and *L*-cystine compared to HEDS. However, the predicted dissociation constant of the *L. japonicus* protein for this substrate remained several orders of magnitude higher than that calculated for *Sy*GRXA (Table 1).

Another interesting feature found for both *Lj*GRX460 and *Lj*GRX569 was their tendency to form soluble high molecular weight aggregate species. This was observed for the *L. japonicus* proteins and for *Sy*GRXA, but not for *Sy*GRXC (Figure 7), suggesting that such aggregates could be generated in any GRX containing more than one cysteine residue, regardless of their class. Similar multimeric behavior has been described for several plant GRXs, including class I, II and III GRXs [17,32], suggesting that the formation of higher order assemblies may be a broader property of GRXs than previously recognized. Although the precise molecular organization of these assemblies remains to be established, their solubility indicates that they do not correspond to irreversible aggregation. In fact, circular dichroism analysis of CC-type GRXs forming high molecular weight species indicated a folded glutaredoxin structure of the oligomers [17]. Nevertheless, changes in GRX oligomeric state may entail a change in protein function, as observed for *At*GRXS17, where the species coordinating the [Fe–S] center is involved in the maturation of cytosolic and nuclear iron–sulfur proteins, whereas its high molecular weight aggregates act as ATP-dependent molecular chaperones that promote disaggregation of the malate dehydrogenase *At*MDH1 [32].

Although *Lj*GRX460 and *Lj*GRX569 predominantly formed high molecular weight soluble assemblies under our experimental conditions, the absence of structural information regarding their quaternary organization precluded reliable modelling of the assembled state. Therefore, the molecular dynamics simulations and docking analyses were performed on monomeric models and should be interpreted as describing the intrinsic structural properties and active site accessibility of the individual protein subunits. Moreover, although oligomerization could influence substrate accessibility *in vivo* or *in vitro*, the catalytic motifs and glutathione-binding residues are intrinsic features of each individual polypeptide. Therefore, the analyses of the monomeric structures carried out remain informative for the study of the features of the active site and for comparing *Lj*GRX460 and *Lj*GRX569 proteins with previously characterized GRXs. Future structural studies will be required to determine how oligomerization influences their biochemical properties.

The low redox activity measured with different substrates for *Sy*GRXC, *Lj*GRX460 and *Lj*GRX569, together with the structural data presented, suggests the possibility that these *Lj*GRXs may act *in vivo* through interaction with other proteins. Yeast two-hybrid assays have shown that most class III GRXs from *A. thaliana* could interact with TGACG-binding (TGA) transcription factors and/or with transcriptional co-repressors of the TOPLESS family [5]. Among the TGA transcription factors analyzed, one of the most frequently identified was TGA2 [5], a salicylic acid-responsive and NPR (nonexpressor of pathogen related genes)-dependent transcriptional activator. NPR1 is present as an oligomer of monomers linked by intermolecular disulfides, but following changes in the cellular redox potential led to a monomeric form that accumulates in the nucleus, promoting disassembly of TGA2 oligomers to activate the transcription of target genes [33,34]. A similar regulatory mechanism has been described for *Lj*FUN (fixation under nitrate), a transcription factor of the TGA family in *L. japonicus*, which forms oligomers that acquire filamentous structures and, depending on zinc availability in the medium, transitions to a monomeric state that promotes the induction of nodule senescence genes [35].

## 4. Materials and Methods

### 4.1. Sequence and Phylogenetic Analyses

Phylogenetic trees were constructed using protein sequences. Multiple sequence alignment was performed in MEGA v12.0.11 [36] using MUSCLE [37]. Model selection was made under a maximum likelihood framework using up to five parallel computing threads. The optimal substitution model was chosen based on the lowest Bayesian Information Criterion. Phylogenetic inference was then conducted using the maximum likelihood method with the selected model, 1000 bootstrap replicates and partial deletion to remove alignment positions with site coverage below 95%.

### 4.2. Structural Modelling and Molecular Dynamics

Structural models of *Lj*GRX460, *Lj*GRX569 and *Sy*GRXC were generated by comparative (template-based) modelling using MODELLER v10.6 [38]. Suitable templates were identified in the PDB [39] by BLAST v 2.17.0 sequence searches using the BLOSUM62 substitution matrix [40] (Table S2), and crystallographic water molecules from the templates were retained. For each target, 100–250 models were built and ranked by normalized discrete optimized protein energy (zDOPE) [41], RMSD and template overlap. Model quality was evaluated with WHAT_CHECK v8.1, PROCHECK v3.5.4 and VERIFY3D v1.0 via the SAVES v6.1 server (https://saves.mbi.ucla.edu/, accessed on 10 September 2025) [42,43,44]. Molecular dynamics simulations were also performed on the CICA (Centro Informático Científico de Andalucía) high-performance computing (Universidad de Sevilla) system using AMBER24 v3.0 [45] with the ff19SB force field [46] under periodic boundary conditions in an orthorhombic box. Systems were solvated with optimal point charge (OPC) water [47] and neutralized with counterions, subjected to staged energy minimization (protein restrained, then solvent/ions restrained), equilibrated for 400 ps in the isothermal-isobaric (NPT) ensemble at 1 atm (adjusting box volume to a solvent density of 1.03 g·cm^−3^), then minimized and equilibrated at 298 K in the canonical (NVT) ensemble for 400 ns before production trajectories were collected under identical conditions. Trajectories were analyzed with CPPTRAJ v6 [48], and structure manipulation and figures were prepared using Chimera v1.19 software [20].

### 4.3. Protein Expression and Purification

#### 4.3.1. LjGRXs

*Lj*GRX460 and *Lj*GRX569 DNA coding sequences were cloned in frame to an amino-terminal Strep-tag II in the pET51b(+) (Novagen, Merck KGaA, Darmstadt, Germany) plasmid using BamHI-SacI or BamHI-HindIII restriction sites, respectively. These modified plasmids were used to transform *E. coli* BL21(DE3)pLysS strains, which were grown at 37 °C and 120 rpm until reaching an OD_600_ of 1. Protein expression was induced with 0.2 mM IPTG, and cultures were incubated at 20 °C for 72 h. Cells from 1 L of culture were harvested and resuspended in 5 mL of lysis buffer (containing 100 mM Tris-HCl (pH 8.0), 150 mM NaCl, 1 mM EDTA) per g of fresh weight. Cells were broken by sonication in the presence of 1 mM DTT, 0.1% (*v*/*v*) Tween 20 and 1 mM PMSF through a FB120 sonicator (Fisher Scientific, Alcobendas, Madrid, Spain) during 40 min, with cycles of 1 s sonication (20% amplitude)/1 s pause. Cell debris was removed by centrifugation at 3000 g for 20 min at 4 °C. The liquid supernatant was filtered with a 0.7 μM filter and applied to 1 mL of Strep-Tactin^®^ Superflow^®^ high-capacity resin (IBA Lifesciences GmbH, Göttingen, Germany). Crude extract and resin were incubated overnight at 4 °C under gentle rotation. Flow-through was collected, and resin was washed with 15 bed volumes of lysis buffer. Bound proteins were eluted in 6 fractions (0.5 bed volumes each) with elution buffer (100 mM Tris-HCl (pH 8.0), 150 mM NaCl, 1 mM EDTA and 2.5 mM desthiobiotin) containing 1 mM DTT and 0.1% (*v*/*v*) Tween 20. Buffer exchange of eluted fractions to 100 mM Tris-HCl (pH 8.0) and 2 mM EDTA was performed through PD-10 desalting columns (Sephadex^TM^ G-25, Cytiva, Marlborough, MA, USA), following a strategy commonly employed to obtain reduced proteins while eliminating low molecular weight thiols before biochemical characterization [17,49]. Samples obtained were concentrated with Vivaspin^®^ (Sartorius AG, Göttingen, Germany) centrifugal filters with a 5 kDa molecular-weight cut-off.

#### 4.3.2. SyGRXs

Recombinant *Sy*GRXs were obtained with an amino-terminal 6×His-tag from *E. coli* DH5α strains previously transformed with modified pQE80 plasmids generated by López-Maury et al. [50]. Protein expression was induced following conditions previously described [50]. Cells from 200 mL of culture were harvested and resuspended in 5 mL of lysis buffer (containing 50 mM Tris-HCl (pH 8.0) and 0.3 M NaCl) per g of fresh weight. They were broken by sonication like *Lj*GRXs, until reaching 80–90% of lysis, and cell debris was removed by centrifugation at 3000 g for 20 min at 4 °C. Liquid supernatant was filtered with a 0.7 μM filter and applied to 1 mL of Protino^®^ Ni-NTA agarose resin (Macherey-Nagel, Düren, Germany). Purification was carried out according to the manufacturer’s instructions. Bound proteins were eluted in buffers containing increasing imidazole concentrations (50–300 mM), which were subsequently removed through PD-10 desalting columns (Sephadex^TM^ G-25, Cytiva, Marlborough, MA, USA) run in 100 mM Tris-HCl (pH 8.0) and 2 mM EDTA.

### 4.4. Protein Electrophoresis

#### 4.4.1. Denaturing Electrophoresis (SDS-PAGE)

Samples were mixed with 4× buffer (containing 125 mM Tris-HCl (pH 6.8), 20% (*v*/*v*) glycerol, 4% (*v*/*v*) SDS, 10% (*v*/*v*) β-mercaptoethanol and 0.05% (*v*/*v*) bromophenol blue) and boiled at 100 °C for 5 min before loading them in minigels composed of 4% (*w*/*v*) acrylamide stacking gel and 15% (*w*/*v*) acrylamide resolving gel. Electrophoresis was performed in 25 mM Tris, 0.19 M glycine and 0.1% (*v*/*v*) SDS, using a Mini-Protean^®^ Tetra Vertical Electrophoresis Cell system (Bio-Rad, Hercules, CA, USA) at a constant voltage of 200 V with a current limit of 135 mA for 40 min.

#### 4.4.2. Non-Denaturing Electrophoresis

Samples were mixed with 4× buffers like those used for SDS-PAGE, but with different combinations of 1% (*w*/*v*) SDS and 2.5% (*v*/*v*) β-mercaptoethanol. Protein samples mixed with these buffers were loaded in minigels composed of 4% (*w*/*v*) acrylamide stacking gel and 8% (*w*/*v*) acrylamide resolving gel. Electrophoresis was performed in 50 mM Tris and 0.38 M glycine, using a Mini-Protean^®^ Tetra Vertical Electrophoresis Cell system (Bio-Rad, Hercules, CA, USA) at a constant voltage of 20 V with a current limit of 5 mA for 5 h.

### 4.5. Glutaredoxin Oxidoreductase Activity Assay

The oxidoreductase activity of recombinant GRXs was determined using a glutathione reductase (GR) coupled assay adapted from the specifications in [51]. The GSSG generated during the GRX-catalyzed reaction was reduced back to GSH by GR through an NADPH-dependent process, which was monitored for 5 min according to the decrease in the absorbance at 340 nm using a Shimadzu UV-1280 UV–Vis spectrophotometer (Shimadzu Corporation. Kyoto, Japan). Reactions were performed in quartz cuvettes, with a final volume of 1 mL containing 100 mM Tris-HCl (pH 8.0), 2 mM EDTA, 0.2 mM NADPH, either 0.7 mM HEDS or 2.5 mM *L*-cystine (prepared in 1 N HCl), 1 mM GSH, 0.2–2 μM GRX and 4.5 μg/mL GR, which was added last. Enzymatic activity was calculated using the molar extinction coefficient of NADPH (ε_340_ = 6.22 mM^−1^·cm^−1^) and subtracting the absorbance of the corresponding controls without GRX.

### 4.6. Molecular Docking

Docking calculations were carried out to assess binding of HEDS and *L*-cystine to the *Sy*GRXA and *Lj*GRX460 models using the Lamarckian genetic algorithm implemented in AutoDockTools v4.2 [52,53]. Ligand partial charges were assigned by the Gasteiger method using Antechamber from AmberTools v24 [54]. Protein charges were assigned according to the AMBER99SB force field. An initial blind docking over the full protein surface was followed by flexible docking in which selected binding-site side chains were treated as flexible (Table S3).

## 5. Conclusions

The present study provides a comprehensive molecular characterization of two symbiosis-responsive CC-type GRXs from *L. japonicus*, combining comparative sequence analysis, structural modelling, recombinant protein production and biochemical characterization. Although *Lj*GRX460 and *Lj*GRX569 retain the characteristic CC-type active site, they exhibit structural and biochemical features that clearly distinguish them from canonical class I GRXs. Their limited oxidoreductase activity towards classical disulfide substrates, together with reduced substrate accessibility predicted by structural modelling of the monomeric proteins and docking analyses, supports the view that these proteins are not primarily dedicated to thiol–disulfide oxidoreduction. The successful production of soluble recombinant proteins and the observation that both proteins form stable high molecular weight species also provide valuable experimental tools and new insights into the molecular properties of plant CC-type GRXs. Given that both proteins were originally identified as symbiosis-responsive genes, these findings establish a foundation for future studies aimed at identifying their physiological interaction partners and elucidating their role in the signaling networks regulating legume–rhizobia symbiosis and biological nitrogen fixation.

## Figures and Tables

**Table 1:** **Flexible targeted docking of *Lj*GRX460 and *Sy*GRXA with HEDS and *L*-cystine**. Mean dissociation constant (K_D_) and mean association free energy (ΔG_A_°) of the lowest energy cluster (upper part). Dissociation constant (K_D_) and energy terms (ΔG_A_°: association free energy; E_T_: torsional energy; E_E_: electrostatic energy; E_VdW+HB+Desolv_: energy due to van der Waals interactions, hydrogen bonding and desolvation; E_I_: intermolecular energy) of the most favourable docked conformation within the lowest energy cluster (bottom part).

	*Lj*GRX460		*Sy*GRXA	
	HEDS	*L*-cystine	HEDS	*L*-cystine
**K_D_ (μM)**	3.63 × 10^3^	45.65	720.89	1.55
**ΔG_A_° (kcal/mol)**	−3.34	−5.94	−4.30	−7.95
				
	***Lj*** **GRX460**		***Sy*** **GRXA**	
	**HEDS**	***L*** **-cystine**	**HEDS**	***L*** **-cystine**
**K_D_ (μM)**	315.35	40.58	27.22	7.64 × 10^−3^
**Δ** **G_A_° (kcal/mol)**	−4.78	−5.99	−6.23	−11.07
**E_T_ (kcal/mol)**	2.09	2.68	2.09	2.68
**E_E_ (kcal/mol)**	−0.77	−2.74	−0.85	−5.17
**E_VdW + HB + Desolv_ (** **kcal/mol)**	−6.09	−5.94	−7.46	−8.59
**^1^** **E_I_ (kcal/mol)**	−8.68	−8.57	−8.32	−13.76

**^1^E_I_ = E_E_ + E_VdW+HB+Desolv_**.

## Data Availability

All data generated or analyzed during this study are included in the published article and its Supplementary Materials.

## Supplementary Materials

The following supporting information can be downloaded at: https://www.mdpi.com/article/10.3390/ijms27156703/s1.

## Abbreviations

The following abbreviations are used in this manuscript:

GRX	glutaredoxin
HEDS	bis(2-hydroxyethyl) disulfide
TRX	thioredoxin
GSH	reduced glutathione
ROXYs	class III glutaredoxins
RMSD	root mean square deviation
DSSP	dictionary of protein secondary structure
PDB	protein data bank
IPTG	isopropyl β-D-1-thiogalactopyranoside
OD_600_	optical density at 600 nm wavelength
PAGE	polyacrylamide gel electrophoresis
SDS	sodium dodecyl sulfate
MBP	maltose-binding protein
GST	glutathione S-transferase
GSSG	oxidized glutathione
TGA	TGACG-binding transcription factor
NPR	nonexpressor of pathogen related genes
FUN	fixation under nitrate
zDOPE	normalized discrete optimized protein energy
OPC	optimal point charge
NPT	isothermal-isobaric ensemble
NVT	canonical ensemble
Tris	tris(hydroxymethyl)aminomethane
EDTA	ethylenediaminetetraacetic acid
DTT	dithiothreitol
Tween 20	polyoxyethylene (20) sorbitan monolaurate
PMSF	phenylmethylsulfonyl fluoride
GR	glutathione reductase
