# Supplementary material for: Molecular Analysis of Two CC-Type Glutaredoxins from the Model Legume Lotus japonicus
Source: Int J Mol Sci. 2026 Jul 27;27(15):6703. doi: 10.3390/ijms27156703 (PMC13466483; doi:10.3390/ijms27156703)
Supplement: Supplementary file 1 [file ijms-27-06703-s001.zip › Supplementary Materials.pdf]

Supplementary Materials

Table S1. **Sequence identity analyses.** Percentage identity of protein (aa) and coding sequences (CDS) of *Lj*GRX460 and *Lj*GRX569 relative to *At*GRXs.

	*At*GRX		% Identity (aa)		% Identity (CDS)	
	**Common name**	**ID transcript**	***Lj*GRX460**	***Lj*GRX569**	***Lj*GRX460**	***Lj*GRX569**
**CLASS I**	*At5g63030.1*	*At*GRXC1	36.78	38.24	49.80	50.55
	At5g40370.1	*At*GRXC2.1	37.21	34.65	47.04	46.15
	At5g40370.2	*At*GRXC2.2	35.63	30.39	45.45	42.12
	At1g77370.1	*At*GRXC3.1	31.03	28.87	36.84	41.70
	At1g77370.2	*At*GRXC3.2	31.03	28.87	35.21	40.07
	At5g20500.1	*At*GRXC4	33.33	33.33	44.92	41.49
	At4g28730.1	*At*GRXC5.1	34.48	36.27	46.61	47.16
	At4g28730.2	*At*GRXC5.2	36.62	40.28	47.87	49.48
	At2g20270.1	*At*GRXS12.1	39.08	36.27	48.05	48.01
	At2g20270.2	*At*GRXS12.2	39.08	36.27	43.82	45.51
**CLASS II**	*At3g54900.1*	*At*GRXS14	21.43	23.71	36.47	40.00
	At3g15660.1	*At*GRXS15.1	34.48	24.24	38.32	34.63
	At3g15660.2	*At*GRXS15.2	36.62	24.24	38.32	34.63
	At2g38270.1	*At*GRXS16	20.69	26.32	27.78	31.90
	At4g04950.1	*At*GRXS17	19.05	20.43	38.20	34.32
**CLASS III**	*At3g02000.1*	*At*ROXY1/*At*GRXC7	44.32	57.84	50.19	58.65
	At5g14070.1	*At*ROXY2/*At*GRC8	45.45	53.40	52.06	57.37
	At3g21460.1	*At*ROXY3/*At*GRXS10	42.05	62.75	54.31	61.17
	At3g62950.1	*At*ROXY4/*At*GRXC11	45.45	50.49	52.43	57.37
	At2g47870.1	*At*ROXY5/*At*GRXC12	43.18	54.37	52.81	60.26
	At1g06830.1	*At*ROXY6/*At*GRXS11	45.45	55.56	55.43	57.67
	At2g30540.1	*At*ROXY7/*At*GRXS9	46.59	54.90	57.30	57.28
	At3g62960.1	*At*ROXY8/*At*GRXC14	44.32	49.02	54.68	54.05
	At2g47880.1	*At*ROXY9/*At*GRXC13	46.59	50.98	56.18	57.28
	At5g18600.1	*At*ROXY10/*At*GRXS2	54.55	83.33	61.42	75.08
	At4g15700.1	*At*ROXY11/*At*GRXS3	48.86	75.49	62.17	72.49
	At4g15690.1	*At*ROXY12/*At*GRXS5	50.00	77.45	60.67	70.23

At4g15680.1	*At*ROXY13/*At*GRXS4	51.14	75.49	61.80	70.55
At4g15670.1	*At*ROXY14/*At*GRXS7	47.73	75.49	61.42	70.55
At4g15660.1	*At*ROXY15/*At*GRXS8	48.86	74.51	61.42	70.23
At1g03020.1	*At*ROXY16/*At*GRXS1	56.82	57.84	65.92	61.49
At3g62930.1	*At*ROXY17/*At*GRXS6	62.50	57.84	69.29	61.17
At1g03850.1	*At*ROXY18.1/*At*GRXS13.1	27.50	33.72	47.66	43.68
At1g03850.2	*At*ROXY18.2/AtGRXS13.2	27.50	40.45	47.23	48.74
At1g28480.1	*At*ROXY19/*At*GRXC9	28.24	43.16	46.90	49.33
At5g11930.1	*At*ROXY20/*At*GRXC10	34.62	45.88	49.78	51.30
At4g33040.1	*At*ROXY21/*At*GRXC6	35.71	44.57	48.99	49.49

Class	Motif	Protein	Sequence	Length
Class I	CXXC	*At*GRXC1	--	0
		*At*GRXC2.1	--	0
		*At*GRXC2.2	--	0
		*At*GRXC3.1	--	0
		*At*GRXC3.2	--	0
		*At*GRXC4	--	0
		*At*GRXC5.1	--	0
		*At*GRXC5.2	--	0
		*Sy*GRXA	--	0
		*Sy*GRXB	--	0
		*At*GRXS12.1	--	0
		*At*GRXS12.2	--	0
Class III	CCXC	*At*ROXY1/*At*GRXC7	--	0
		*At*ROXY2/*At*GRC8	--	0
		*At*ROXY4/*At*GRXC11	--	0
		*At*ROXY5/*At*GRXC12	--	0
		*At*ROXY8/*At*GRXC14	--	0
		*At*ROXY9/*At*GRXC13	--	0
		*At*ROXY19/*At*GRXC9	--	0
		*At*ROXY20/*At*GRXC10	--	0
		*At*ROXY21/*At*GRXC6	--	0
		*Lj*GRX460	--	0
	CCXX	*Lj*GRX569	--	0
		*At*ROXY3/*At*GRXS10	--	0
		*At*ROXY6/*At*GRXS11	--	0
		*At*ROXY7/*At*GRXS9	--	0
		*At*ROXY10/*At*GRXS2	--	0
		*At*ROXY11/*At*GRXS3	--	0
		*At*ROXY12/*At*GRXS5	--	0
		*At*ROXY13/*At*GRXS4	--	0
		*At*ROXY14/*At*GRXS7	--	0
		*At*ROXY15/*At*GRXS8	--	0
		*At*ROXY16/*At*GRXS1	--	0
		*At*ROXY17/*At*GRXS6	--	0
		*At*ROXY18.1/*At*GRXS13.1	--	0
		*At*ROXY18.2/*At*GRXS13.2	--	0
Class II		*At*GRXS14	--MALRSVKTPTLITSVAVVSSS---VTNKPHSI	29
		*At*GRXS15.1	--	0
		*At*GRXS15.2	--	0
		*At*GRXS16	---MAAIT---------ISSSL	10
		*At*GRXS17	MSGTVKDIVSKAELDNLRQSGAPVVLHFWASWCDASKQMDQVFSHLATDFPRAHFFRVEAEEHPEISEAYSVAAVPYFVFFKDGKTVDTLEGADPSSLANKVGKVAGSSTSAEPAAPASL	120
		*Sy*GRXC	--	0

```
Class I    CXXC  AtGRXC1                 ------------------------------------------------------------------------------------------------------------------------   0
                 AtGRXC2.1               ------------------------------------------------------------------------------------------------------------------------   0
                 AtGRXC2.2               ------------------------------------------------------------------------------------------------------------------------   0
                 AtGRXC3.1               ------------------------------------------------------------------------------------------------------------------------   0
                 AtGRXC3.2               ------------------------------------------------------------------------------------------------------------------------   0
                 AtGRXC4                 ------------------------------------------------------------------------------------------------------------------------   0
                 AtGRXC5.1               ------------------------------------------------------------------------------------------------------------------------   0
                 AtGRXC5.2               ------------------------------------------------------------------------------------------------------------------------   0
                 SyGRXA                  ------------------------------------------------------------------------------------------------------------------------   0
                 SyGRXB                  ------------------------------------------------------------------------------------------------------------------------   0
                 AtGRXS12.1              ------------------------------------------------------------------------------------------------------------------------   0
                 AtGRXS12.2              ------------------------------------------------------------------------------------------------------------------------   0

Class III  CCXC  AtROXY1/AtGRXC7         ------------------------------------------------------------------------------------------------------------------------   0
                 AtROXY2/AtGRC8          ------------------------------------------------------------------------------------------------------------------------   0
                 AtROXY4/AtGRXC11        ------------------------------------------------------------------------------------------------------------------------   0
                 AtROXY5/AtGRXC12        ------------------------------------------------------------------------------------------------------------------------   0
                 AtROXY8/AtGRXC14        ------------------------------------------------------------------------------------------------------------------------   0
                 AtROXY9/AtGRXC13        ------------------------------------------------------------------------------------------------------------------------   0
                 AtROXY19/AtGRXC9        ------------------------------------------------------------------------------------------------------------------------   0
                 AtROXY20/AtGRXC10       ------------------------------------------------------------------------------------------------------------------------   0
                 AtROXY21/AtGRXC6        ------------------------------------------------------------------------------------------------------------------------   0
                 LjGRX460                ------------------------------------------------------------------------------------------------------------------------   0

           CCXX  LjGRX569                ------------------------------------------------------------------------------------------------------------------------   0
                 AtROXY3/AtGRXS10        ------------------------------------------------------------------------------------------------------------------------   0
                 AtROXY6/AtGRXS11        ------------------------------------------------------------------------------------------------------------------------   0
                 AtROXY7/AtGRXS9         ------------------------------------------------------------------------------------------------------------------------   0
                 AtROXY10/AtGRXS2        ------------------------------------------------------------------------------------------------------------------------   0
                 AtROXY11/AtGRXS3        ------------------------------------------------------------------------------------------------------------------------   0
                 AtROXY12/AtGRXS5        ------------------------------------------------------------------------------------------------------------------------   0
                 AtROXY13/AtGRXS4        ------------------------------------------------------------------------------------------------------------------------   0
                 AtROXY14/AtGRXS7        ------------------------------------------------------------------------------------------------------------------------   0
                 AtROXY15/AtGRXS8        ------------------------------------------------------------------------------------------------------------------------   0
                 AtROXY16/AtGRXS1        ------------------------------------------------------------------------------------------------------------------------   0
                 AtROXY17/AtGRXS6        ------------------------------------------------------------------------------------------------------------------------   0
                 AtROXY18.1/AtGRXS13.1   ------------------------------------------------------------------------------------------------------------------------   0
                 AtROXY18.2/AtGRXS13.2   ------------------------------------------------------------------------------------------------------------------------   0

Class II         AtGRXS14                RFSLKPTSALVVHNHQLSFYGSNLKLKPTKFRCSASALTPQLK-----------------------------------------------------------------------------  72
                 AtGRXS15.1              ------------------------------------------------------------------------------------------------------------------------  0
                 AtGRXS15.2              ------------------------------------------------------------------------------------------------------------------------  0
                 AtGRXS16                HASASPRVVRPHVS-------RNTPVITLYSRFTPSFSFPSLSFTLRD------------TAPSRRRSFFIASAVKSLTETE-------LLPI-------TEADSIPSASGVYAV-----  92
                 AtGRXS17                GLAAGPTILETVKE--------NAK-ASLQDRAQPVSTADALKSRLEKLTNSHPVMLFMKGIPEEPRCGFSRKVVDILKEVNVDFGSFDILSDNEVREGLKKFSNWPTFPQLYCNGELLG  231
                 SyGRXC                  -------------------------------------MNPETK-----------------------------------------------------------------------------  6
```

```
Class I   CXXC  AtGRXC1                  ----------------------------------------------------------------------------------------------------------MGSMFSG-------  7
                AtGRXC2.1                ------------------------------------------------------------------------------------------------------------------------  0
                AtGRXC2.2                ----------------------------------------------------------------------------------------------------------MDSIMCVSSW----  10
                AtGRXC3.1                ------------------------------------------------------------------------------------------------------------MVDQSPRRVVVA  12
                AtGRXC3.2                ------------------------------------------------------------------------------------------------------------MVDQSPRRVVVA  12
                AtGRXC4                  ----------------------------------------------------------------------------------------------------------MTMFRSISMVMLLV  14
                AtGRXC5.1                --------------------------------------------MAVTAFNTLKLVSSSLDPIPSVSC---------------------------SSYSFSLIYVGSPYKRCLKQSCSVR  49
                AtGRXC5.2                --------------------------------------------MAVTAFNTLKLVSSSLDPIPSVSC---------------------------SSYSFSLIYVGSPYKRCLKQSCSVR  49
                SyGRXA                   ------------------------------------------------------------------------------------------------------------------------  0
                SyGRXB                   ------------------------------------------------------------------------------------------------------------------------  0
                AtGRXS12.1               --------------------------------------------MVAATVNLANMTWTSLNSNPAISF---------------------------SMLSGIRNLGMLPFRRCLKPTVIGI  49
                AtGRXS12.2               --------------------------------------------MVAATVNLANMTWTSLNSNPAISF---------------------------SMLSGIRNLGMLPFRRCLKPTVIGI  49

Class III CCXC  AtROXY1/AtGRXC7          ------------------------------------------------------------------------------------------------------------MQYQTESW----  8
                AtROXY2/AtGRC8           ------------------------------------------------------------------------------------------------------------MQYKTETR----  8
                AtROXY4/AtGRXC11         ------------------------------------------------------------------------------------------------------------------------  0
                AtROXY5/AtGRXC12         ------------------------------------------------------------------------------------------------------------------------  0
                AtROXY8/AtGRXC14         ------------------------------------------------------------------------------------------------------------------------  0
                AtROXY9/AtGRXC13         ------------------------------------------------------------------------------------------------------------------------  0
                AtROXY19/AtGRXC9         ------------------------------------------------------------------------------------------------------------MQGTI-------  5
                AtROXY20/AtGRXC10        ------------------------------------------------------------------------------------------------------MKTMRGLRNCSNDAVTLD  18
                AtROXY21/AtGRXC6         ------------------------------------------------------------------------------------------------------MMQELGLQRFSNDVVRLD  18
                LjGRX460                 ------------------------------------------------------------------------------------------------------------------------  0

          CCXX  LjGRX569                 ------------------------------------------------------------------------------------------------------------------------  0
                AtROXY3/AtGRXS10         ------------------------------------------------------------------------------------------------------------------------  0
                AtROXY6/AtGRXS11         ------------------------------------------------------------------------------------------------------------------------  0
                AtROXY7/AtGRXS9          ------------------------------------------------------------------------------------------------------------------------  0
                AtROXY10/AtGRXS2         ------------------------------------------------------------------------------------------------------------------------  0
                AtROXY11/AtGRXS3         ------------------------------------------------------------------------------------------------------------------------  0
                AtROXY12/AtGRXS5         ------------------------------------------------------------------------------------------------------------------------  0
                AtROXY13/AtGRXS4         ------------------------------------------------------------------------------------------------------------------------  0
                AtROXY14/AtGRXS7         ------------------------------------------------------------------------------------------------------------------------  0
                AtROXY15/AtGRXS8         ------------------------------------------------------------------------------------------------------------------------  0
                AtROXY16/AtGRXS1         ------------------------------------------------------------------------------------------------------------------------  0
                AtROXY17/AtGRXS6         ------------------------------------------------------------------------------------------------------------------------  0
                AtROXY18.1/AtGRXS13.1    ------------------------------------------------------------------------------------------------------------MQKAI-------  5
                AtROXY18.2/AtGRXS13.2    ------------------------------------------------------------------------------------------------------------MQKAI-------  5

Class II        AtGRXS14                 ------------------------------------------------------------------------------------------------------------------------  72
                AtGRXS15.1               ---------------------------------------------------MAASLSSRL--------IKGIA-------------------------NL---KAVRS-SR-LTSASVYQ  31
                AtGRXS15.2               ---------------------------------------------------MAASLSSRL--------IKGIA-------------------------NL---KAVRS-SR-LTSASVYQ  31
                AtGRXS16                 -------YDKS----DELQF------------------------VGISRNIAASVSAHLKSV----------P--ELCGSV-----------------KVGIVEEP-DKAVLTQAWKLWI  147
                AtGRXS17                 GADIAIAMHESGELKDAFKDLGITTVGSKESQDEAGKGGGVSSGNTGLSETLRARLEGLVNSKPVMLFMKGRPEEPKCGFSGKVVEILNQEKIEFGSFDILLDDEVRQGLKVYSNWSSYP  351
                SyGRXC                   ------------------------------------------------------------------------------------------------------------------------  6
```

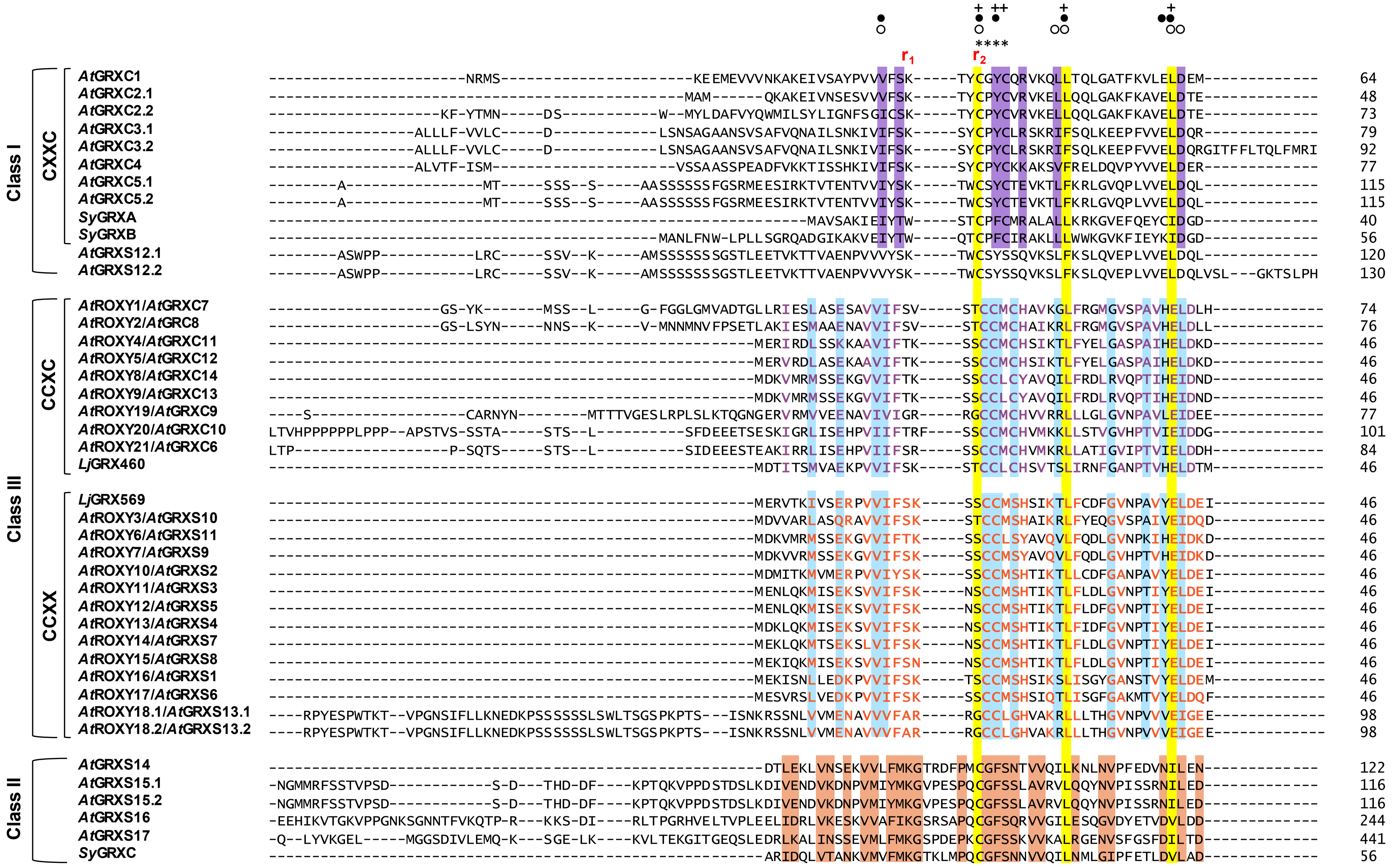

Class I
CXXC
AtGRXC1
AtGRXC2.1
AtGRXC2.2
AtGRXC3.1
AtGRXC3.2
AtGRXC4
AtGRXC5.1
AtGRXC5.2
SyGRXA
SyGRXB
AtGRXS12.1
AtGRXS12.2
Class III
CCXC
AtROXY1/AtGRXC7
AtROXY2/AtGRC8
AtROXY4/AtGRXC11
AtROXY5/AtGRXC12
AtROXY8/AtGRXC14
AtROXY9/AtGRXC13
AtROXY19/AtGRXC9
AtROXY20/AtGRXC10
AtROXY21/AtGRXC6
LjGRX460
CCXX
LjGRX569
AtROXY3/AtGRXS10
AtROXY6/AtGRXS11
AtROXY7/AtGRXS9
AtROXY10/AtGRXS2
AtROXY11/AtGRXS3
AtROXY12/AtGRXS5
AtROXY13/AtGRXS4
AtROXY14/AtGRXS7
AtROXY15/AtGRXS8
AtROXY16/AtGRXS1
AtROXY17/AtGRXS6
AtROXY18.1/AtGRXS13.1
AtROXY18.2/AtGRXS13.2
Class II
AtGRXS14
AtGRXS15.1
AtGRXS15.2
AtGRXS16
AtGRXS17
SyGRXC
r1
r2

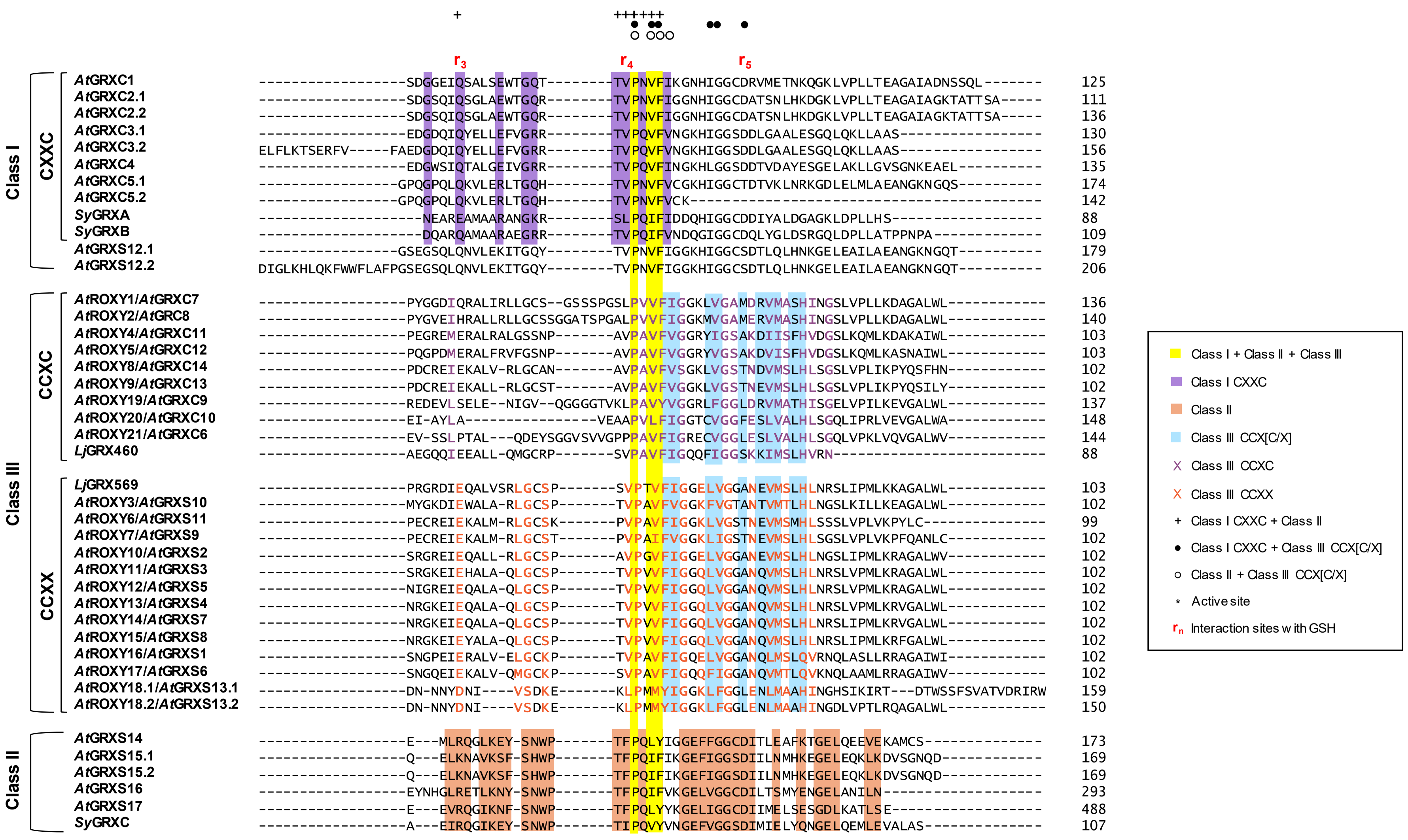
Class I
CXXC
AtGRXC1 125
AtGRXC2.1 111
AtGRXC2.2 136
AtGRXC3.1 130
AtGRXC3.2 156
AtGRXC4 135
AtGRXC5.1 174
AtGRXC5.2 142
SyGRXA 88
SyGRXB 109
AtGRXS12.1 179
AtGRXS12.2 206
Class III
CCXC
AtROXY1/AtGRXC7 136
AtROXY2/AtGRC8 140
AtROXY4/AtGRXC11 103
AtROXY5/AtGRXC12 103
AtROXY8/AtGRXC14 102
AtROXY9/AtGRXC13 102
AtROXY19/AtGRXC9 137
AtROXY20/AtGRXC10 148
AtROXY21/AtGRXC6 144
LjGRX460 88
CCXX
LjGRX569 103
AtROXY3/AtGRXS10 102
AtROXY6/AtGRXS11 99
AtROXY7/AtGRXS9 102
AtROXY10/AtGRXS2 102
AtROXY11/AtGRXS3 102
AtROXY12/AtGRXS5 102
AtROXY13/AtGRXS4 102
AtROXY14/AtGRXS7 102
AtROXY15/AtGRXS8 102
AtROXY16/AtGRXS1 102
AtROXY17/AtGRXS6 102
AtROXY18.1/AtGRXS13.1 159
AtROXY18.2/AtGRXS13.2 150
Class II
AtGRXS14 173
AtGRXS15.1 169
AtGRXS15.2 169
AtGRXS16 293
AtGRXS17 488
SyGRXC 107
r3
r4
r5
Class I + Class II + Class III
Class I CXXC
Class II
Class III CCX[C/X]
Class III CCXC
Class III CCXX
Class I CXXC + Class II
Class I CXXC + Class III CCX[C/X]
Class II + Class III CCX[C/X]
Active site
rn Interaction sites with GSH

Figure S1. Full alignment and conservation analysis of protein sequences from GRXs of different classes and species. Multiple sequence alignment of the GRXs from *A. thaliana*, from *Synechocystis* sp. PCC 6803, *Lj*GRX460 and *Lj*GRX569 performed with Clustal Omega v1.2.4 [16]. Yellow, violet, orange or blue background indicate residues that are conserved in all GRXs analysed, in class I CXXC-type; in class II or in class III CCX[C/X]-type respectively. Similarly, violet letters indicate residues that are conserved in class III CCXC-type GRXs and orange letters residues that are conserved in class III CCXX-type GRXs. Crosses, black circles and unfilled circles indicate residues that are conserved between class I CXXC-type and class II; between class I CXXC-type and class III CCX[C/X]-type; or between class II and class III CCX[C/X] respectively. Residues of the active site are indicated with asterisks and the positions of residues that have been proposed to contact with GSH according to Mrozek *et al*. [17] are marked as r_{1-5}.

(A)

Residues	d (Å)	θ (⁰)
**C21 – C22**	6.267	192.1

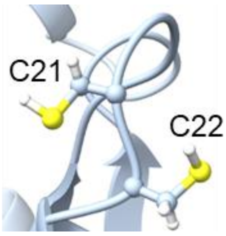

(B)

Residues	d (Å)	θ (⁰)
**C21 – C22**	6.444	315.6
**C21 – C24**	3.627	88.2
**C22 – C24**	5.935	42.5

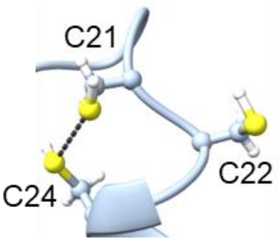

(C)

Residues	d (Å)	θ (⁰)
**C15 – C18**	3.468	73.6
**C15 – C36**	10.413	173.2
**C15 – C70**	8.172	237.1
**C18 – C36**	8.320	96.9
**C18 – C70**	8.152	201.8

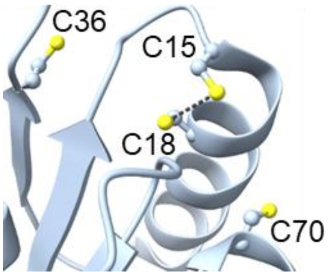

Figure S2. Prediction of intramolecular disulfide bond formation involving active site cysteines. For each GRX (A: *Lj*GRX569; B: *Lj*GRX460; C: *Sy*GRXA) it is shown a table with the distances between sulphur atoms (d) and the angle between the C_β atoms (θ) of the residues involved in the potential disulphide (left) and an enlarged schematic view of the active site region where the most probable interaction leading to an intramolecular disulphide bond formation is indicated with dotted lines (right).

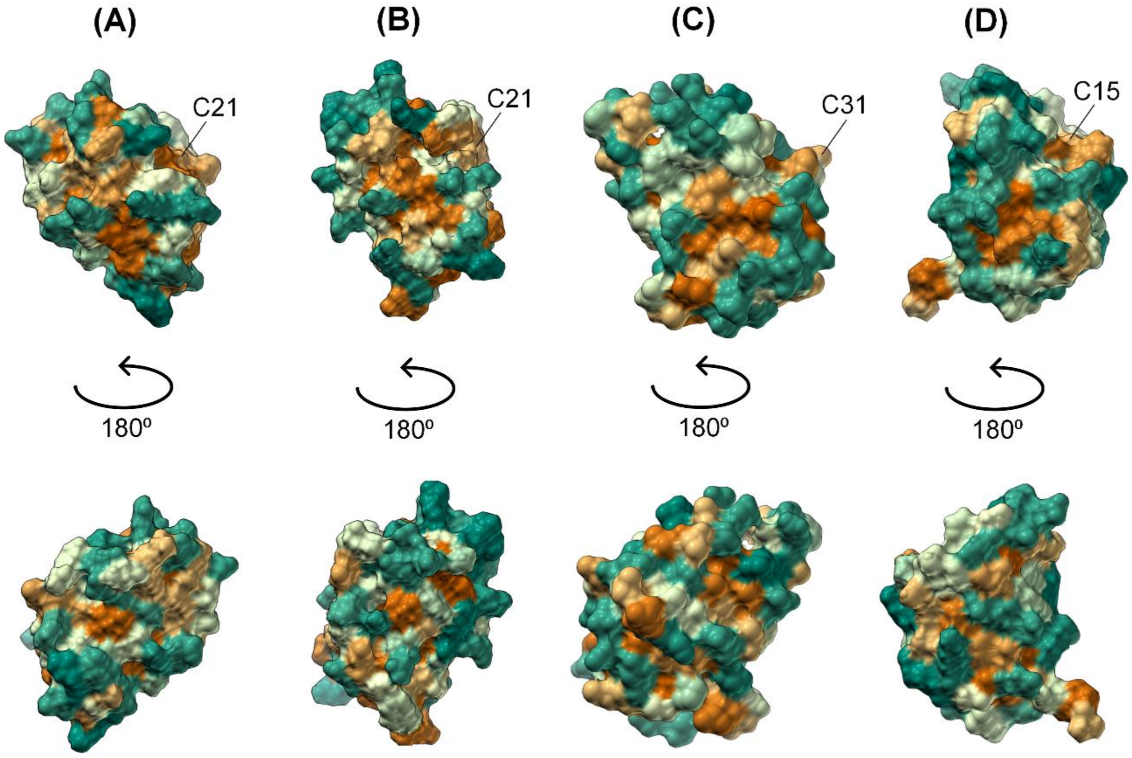

Figure S3. Hydrophobicity of reduced GRXs monomers. For each GRX (A: *Lj*GRX460; B: *Lj*GRX569; C: *Sy*GRXC; D: *Sy*GRXA) it is shown the hydrophobicity of the surface determined using Chimera v1.19 software [20]. Hydrophobic regions are coloured in brown and hydrophilic regions in green. The first cysteine of the active site is indicated.

Table S2. Data of the alignment of GRXs of interest with different templates. For each of the GRX template used is indicated their PDB identifier code, e-value, score and sequence identity percentage (% ID) obtained in the alignment with the GRX of interest, as well as the organism to which they belong.

	**PDB code**	**e-value**	**Score**	**% ID**	**Organism**
	3FZ9	$8.02\ 10^{-15}$	65	37.93	*Populus tremula x tremuloides*
	3FZA	$8.02\ 10^{-15}$	65	37.93	*Populus tremula x tremuloides*
	5KQA	$3.87\ 10^{-13}$	63	40.51	*Eriogonum fasciculatum*
	2FLS	$3.46\ 10^{-13}$	61	34.12	*Homo sapiens*
***Lj*GRX460**	2HT9	$4.23\ 10^{-13}$	61	34.12	*Homo sapiens*
	2MYG	$5.66\ 10^{-13}$	60	36.90	*Trypanosoma brucei*
	5GTX	$6.19\ 10^{-13}$	60	39.24	*Eriogonum fasciculatum*
	5ZVL	$9.47\ 10^{-13}$	61	40.51	*Triticum aestivum*
	3RHB	$4.30\ 10^{-11}$	55	33.33	*Arabidopsis thaliana*
	3FZ9	$1.92\ 10^{-18}$	75	41.41	*Populus tremula x tremuloides*
	5KQA	$3.87\ 10^{-13}$	74	37.00	*Eriogonum fasciculatum*
	2E7P	$9.46\ 10^{-18}$	73	36.36	*Populus tremula x tremuloides*
	1Z7P	$1.21\ 10^{-17}$	73	36.36	*Populus tremula x tremuloides*
***Lj*GRX569**	5GTX	$1.02\ 10^{-16}$	71	36.00	*Eriogonum fasciculatum*
	2FLS	$5.11\ 10^{-15}$	67	35.42	*Homo sapiens*
	2HT9	$6.32\ 10^{-15}$	67	35.42	*Homo sapiens*
	3RHB	$6.91\ 10^{-15}$	66	38.38	*Arabidopsis thaliana*
	5ZVL	$8.64\ 10^{-15}$	67	36.00	*Triticum aestivum*
	3UIW	$7.16\ 10^{-15}$	64	37.63	*Dario rerio*
	2LKU	$2.13\ 10^{-43}$	138	55.14	*Populus trichocarpa*
	2MMA	$1.51\ 10^{-40}$	131	53.27	*Arabidopsis thaliana*
	3IPZ	$1.51\ 10^{-40}$	131	53.27	*Arabidopsis thaliana*
	2WUL	$1.43\ 10^{-38}$	126	58.59	*Homo sapiens*
***Sy*GRXC**	2MMZ	$2.27\ 10^{-38}$	126	58.59	*Homo sapiens*
	2YAN	$1.42\ 10^{-38}$	117	53.61	*Homo sapiens*
	1WIK	$3.76\ 10^{-31}$	107	52.75	*Mus musculus*
	3ZYW	$6.27\ 10^{-30}$	104	47.42	*Homo sapiens*
	1YKA	$1.30\ 10^{-29}$	103	43.14	*Escherichia coli*

Table S3. Flexible residues considered in AutoDock-Flex calculi. Flexible residues considered belonging to active site are highlighted in bold.

Protein	Substrate	Flexible residues
*Sy*GRXA	HEDS	**C15**, **F17**, **C18**, K54, L57, D71
	L-cystine	**C15**, **F17**, **C18**, K54, L57, D71
*Lj*GRX460	HEDS	K18, **C21**, **L23**, **C24**, V65, S78, K79, M82
	L-cystine	K18, **C21**, **L23**, **C24**, V65, S78, K79, M82
